# West Nile virus infection in individuals with pre-existing Usutu virus immunity, northern Italy, 2018

**DOI:** 10.2807/1560-7917.ES.2019.24.21.1900261

**Published:** 2019-05-23

**Authors:** Alessandro Sinigaglia, Monia Pacenti, Thomas Martello, Silvana Pagni, Elisa Franchin, Luisa Barzon

**Affiliations:** 1 Department of Molecular Medicine, University of Padova, Padova, Italy; 2 Microbiology and Virology Unit, Padova University Hospital, Padova, Italy

**Keywords:** West Nile virus, Usutu virus, antibody cross-reactivity, secondary infection, cross-protection

## Abstract

In 2018, there was a large West Nile virus (WNV) outbreak in northern Italy. We observed five atypical cases of WNV infection that were characterised by the presence of WNV RNA and WNV IgG at the time of diagnosis, but no IgM response during follow-up. Neutralisation assays demonstrated pre-existing Usutu virus immunity in all patients. Besides challenging diagnosis, the immunological crosstalk between the two viruses warrants further investigation on possible cross-protection or infection enhancement effects.

During the large West Nile virus (WNV) outbreak that occurred in Northern Italy in 2018, we observed cases with WNV infection and an atypical antibody response, characterised by a strong ‘original antigenic sin’ effect. Here, we demonstrated that these cases of WNV infection occurred in individuals with previous immunity against Usutu virus (USUV), a closely related flavivirus that is also endemic in northern Italy. We describe here the clinical and virological features of these secondary WNV infections and discuss possible clinical implications.

## WNV infection in patients with previous flavivirus immunity

During the surveillance period 1 June–30 November 2018, cases of possible autochthonous arboviral infection were tested at the Regional Reference Laboratory of Veneto Region (Microbiology and Virology Unit, Padova University Hospital, Italy) for confirmation of West Nile virus (WNV) and Usutu virus (USUV) infection according to the surveillance programme of the Italian Ministry of Health [1]. A total of 440 human cases of WNV infection were identified; 81 with neuroinvasive disease, 304 with fever, 34 viraemic blood donors and 21 asymptomatic (or without information on symptoms). In addition, we confirmed eight human cases of USUV infection (one with neuroinvasive disease, six with fever and one viraemic blood donor).

**Figure 1:**
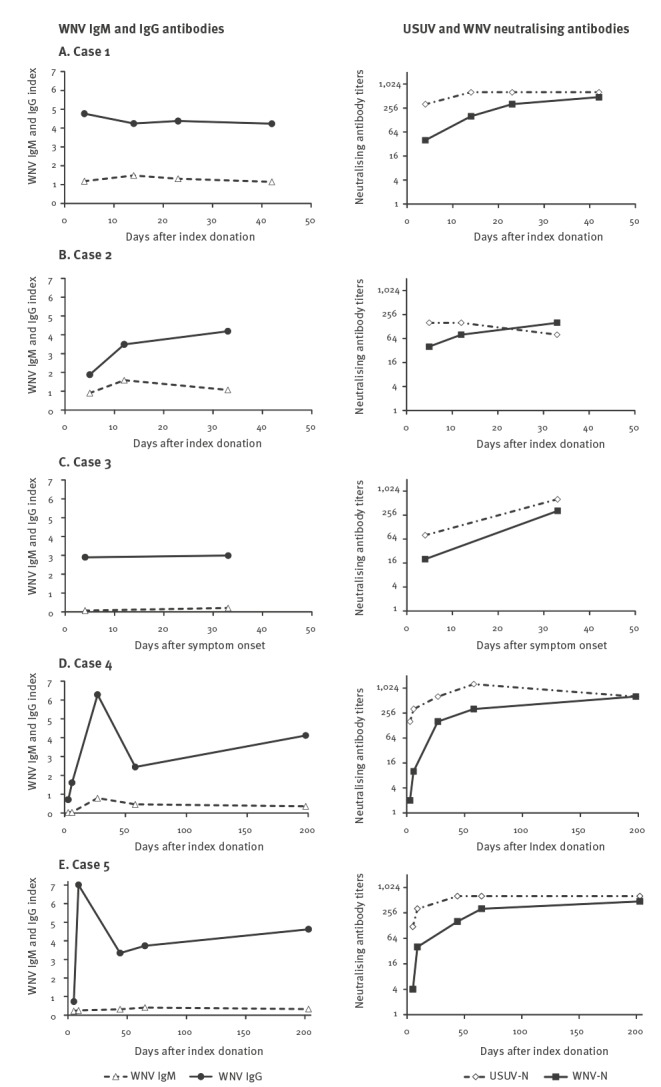
West Nile virus (WNV) IgM and IgG antibodies and Usutu virus and WNV neutralising antibodies in the serum of patients with WNV infection with no specific WNV IgM response, Veneto Region, northern Italy, 2018 (n = 5)

USUV-N: Usutu virus-neutralising antibodies; WNV-N: West Nile virus-neutralising antibodies.

Among individuals with confirmed WNV infection, we observed five cases (four males and one female, median age 51 years, range 32–63 years) that presented with WNV IgG antibodies at the time of diagnosis who lacked a specific IgM antibody response or had a blunted IgM response during follow-up (Figure 1).

These cases included two asymptomatic blood donors (cases 4 and 5), two blood donors – who reported headache, asthenia and myalgia during the previous 2 months (cases 1 and 2) – and a woman in week 28 of pregnancy with rash and asthenia (case 3). The pregnant woman was vaccinated against yellow fever virus in 2004; no other case reported receiving vaccination against any flavivirus nor previous flavivirus infections.

**Figure 2:**
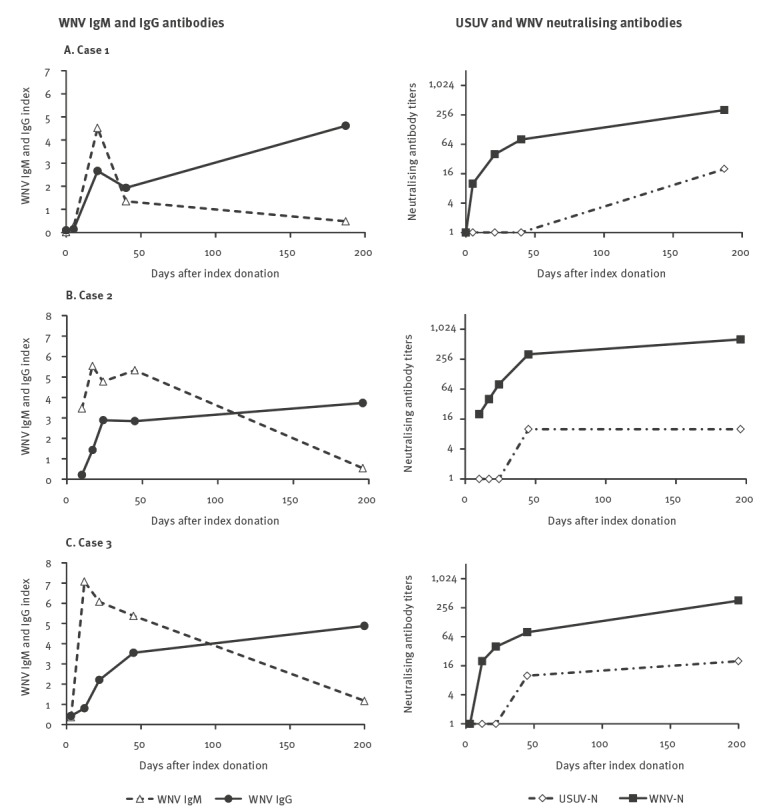
West Nile virus (WNV) IgM and IgG antibodies and Usutu virus and WNV neutralising antibodies in the serum of patients with WNV infection with typical IgM and IgG response following WNV infection, Veneto Region, northern Italy, 2018 (n = 3)

USUV-N: Usutu virus-neutralising antibodies; WNV-N: West Nile virus-neutralising antibodies.

WNV lineage 2 was identified in the two asymptomatic blood donors and in the pregnant woman, while WNV was not typeable in the other cases 1 and 2 because their viral load was too low. In all five cases, WNV RNA was detectable in blood for a short time after index donation or symptom onset (up to 5 days). Neutralisation assays performed at baseline and follow-up serum samples showed low or absent WNV neutralising antibodies and high titres of USUV neutralising antibodies in baseline samples and a progressive increase of WNV neutralisation titres during follow-up, consistent with WNV infection following previous USUV immunity (Figure 1). For comparison, typical IgM and IgG antibody responses following WNV infection are shown in Figure 2. The three cases displayed were asymptomatic blood donors in whom WNV infection was confirmed by molecular testing in 2018.

**Figure 3:**
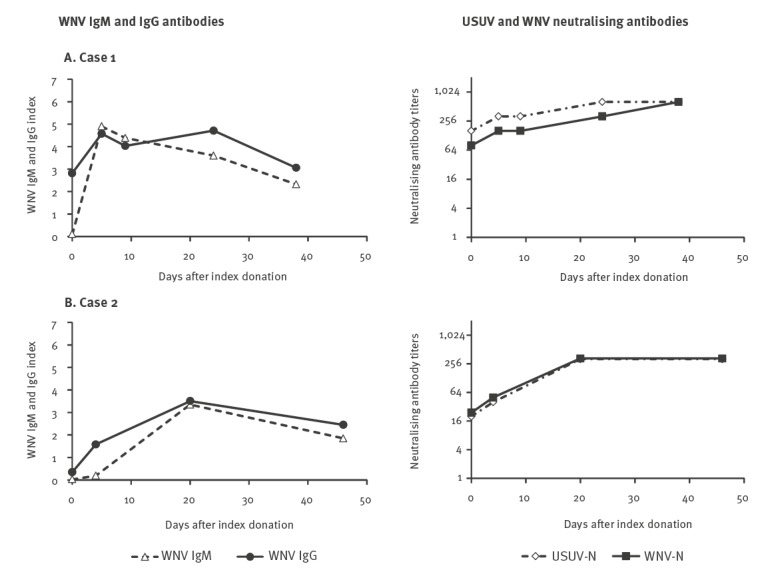
West Nile virus (WNV) IgM and IgG antibodies and Usutu virus and WNV neutralising antibodies in the serum of patients with WNV infection who developed WNV IgM antibody response, with presence of WNV-reactive IgG antibodies at the time of first evaluation, Veneto Region, northern Italy, 2018 (n = 2)

USUV-N: Usutu virus-neutralising antibodies; WNV-N: West Nile virus-neutralising antibodies.

We also observed two cases who were WNV IgM-negative but WNV IgG-positive at the time of first evaluation, who developed high levels of WNV IgM antibodies during follow-up (Figure 3. The cases had detectable WNV RNA in blood at the time of first evaluation, while USUV RNA testing was negative at baseline and during follow-up.

These cases were both asymptomatic women in their 40s, in whom WNV infection was identified by WNV NAT screening for blood donation and cord blood donation, respectively. In both cases, genotyping assay confirmed WNV lineage 2 infection. Case 2 was pregnant (week 39) and WNV RNA was still detectable in whole blood on day 20 after the index donation (i.e. at the time of delivery), while in case 1, WNV RNA was detectable only in the index donation but not in follow-up samples. In both cases, neutralisation assays showed increasing titres of both WNV- and USUV-neutralising antibodies during follow-up, without significant differences between the two viruses (Figure 3). These laboratory findings were consistent with WNV infection in subjects with pre-existing flavivirus immunity, but did not allow identifying the virus responsible for the previous infection. No vaccination against flaviviruses nor previous flavivirus infections were reported by these subjects.

## Laboratory methods

WNV RNA and USUV RNA were detected in whole blood and urine samples by in house real-time RT-PCR methods [2,3]. WNV nucleic acid test (NAT) was performed in plasma samples by using a cobas 6800 system (Roche Molecular Diagnostics, Basel, Switzerland). WNV genotyping was performed by pan-flavivirus nested RT-PCR and sequencing, followed by phylogenetic analysis [2]. WNV IgM -and IgG antibodies were detected in serum by ELISA (WNV IgM capture DxSelect and IgG DxSelect, Focus Diagnostics, Cypress, California, United States). Serum samples with positive results at WNV ELISA were further tested for confirmation by titration of WNV and USUV neutralising antibodies by plaque reduction neutralisation test (PRNT) and microneutralisation titre assay, respectively, on Vero cells [4].

The titres of WNV- and USUV-neutralising antibodies were defined, respectively, as the reciprocal of the highest dilution of the serum that reduced by 50% the number of plaques in Vero cells (PRNT50) and as the reciprocal of the highest dilution of the serum that showed 100% neutralisation of cytopathic effect in microneutralisation assay.

## Discussion

WNV and USUV are genetically related neurotropic mosquito-borne flaviviruses, which are endemic in several European countries [5]. In their transmission cycle, WNV and USUV share the same mosquito vectors and bird populations as amplifying hosts and often the two viruses co-circulate in the same environment [6]. Most WNV infections in humans are asymptomatic or characterised by influenza-like illness, while less than 1% of cases might evolve to severe and potentially fatal neuroinvasive disease, especially in elderly and immunocompromised individuals. USUV appears to be more pathogenic and lethal than WNV for some bird species, while it rarely causes disease in humans [5].

In Italy, the first human cases of WNV infection were detected in northern Italy in 2008 [7]. Since then, outbreaks of WNV infection have occurred every year. Different WNV lineage 1 strains circulated in Italy until 2011, when WNV lineage 2 was first detected and then spread replacing the lineage 1 [8]. The first human cases of USUV infection were detected in northern Italy in 2009 in immunocompromised patients [9,10]. Seroprevalence studies and retrospective investigations suggested that the prevalence of USUV infection in humans could be higher than WNV infection in areas where both viruses co-circulate [11-13]. In 2010, the prevalence of WNV-neutralising antibodies in humans was investigated in 4,450 blood donors and estimated to range between 0.3% and 3% in the different provinces of the Veneto Region [14]; no data are available on USUV seroprevalence. Data are available from the neighbouring Emilia-Romagna Region, where the prevalence of neutralising antibodies against WNV and USUV was evaluated during the same period in 6,000 blood donors and estimated to be 0.78% and 0.23%, respectively [12].

Infections from closely related flaviviruses, like WNV and USUV, may pose problems not only because of the induction of cross-reactive antibodies that challenge the differential diagnosis, but also because the immunological crosstalk between heterologous viruses may increase the risk of severe disease through a mechanism of antibody-dependent enhancement of infection.

The 2018 transmission season recorded a substantial increase in the number of human WNV infections, with ca 1,500 confirmed cases in European Union countries, with Italy as the most affected country [15]. The unprecedented high number of WNV infections recorded in 2018 in the Veneto Region (440 confirmed cases) was conceivably the main factor that led to the identification of the cases of WNV infection with an atypical immune response described here, since the diagnostic process was similar to that of the previous years.

The five cases of WNV infection with atypical immune response were characterised by the presence of WNV RNA in blood and WNV IgG antibodies at the time of diagnosis, but the absence of a specific IgM antibody response or a blunted IgM response during follow-up. All these cases had already USUV neutralising antibodies at the time of the first evaluation and developed WNV neutralising antibodies during follow-up. This pattern of antibody response, which has not been described so far in the literature in patients with WNV infection, is consistent with secondary WNV infection after primary infection with USUV and supports a strong ‘original antigenic sin’ effect between the two viruses. A similar pattern of antibody response has been observed in patients with dengue virus (DENV) infection following infection with a heterologous DENV serotype [16] and in patients with Zika virus infection and previous DENV immunity [17]. It has been associated with cross-protective immunity, as well as with increased risk of severe disease and death through an antibody-dependent enhancement mechanism.

To date, no studies have investigated the immunological interplay between WNV and USUV [18], but experimental studies in mice showed that USUV infection was not lethal in adult mice and conferred cross-protection when infected with a high dose of a WNV neuroinvasive strain [19]. In particular, USUV infection protected mice against WNV disease and death but not against infection, since WNV RNA was detectable 7 days post WNV infection in 50% of USUV pre-infected mice.

Here, the cases of WNV infection that were secondary to USUV infection were asymptomatic or had a mild febrile disease, suggesting that disease enhancement had not occurred. Further investigation including a larger number of cases is needed to better define the clinical and virological features of WNV infection in individuals with pre-existing flavivirus immunity and to understand if USUV infection provides cross-protection against WNV disease or whether it might increase the risk for more severe disease through antibody-dependent enhancement. Finally, two cases of secondary WNV infection were pregnant women who delivered healthy babies: while WNV infection is generally not associated with adverse pregnancy outcome [20], the effect of secondary WNV infection during pregnancy is unknown and warrants investigation. In the context of a considerable increase and expansion of WNV and USUV activity in European countries, clinicians involved in the diagnosis and management of WNV and USUV infections should keep in mind this uncommon picture that challenges laboratory diagnosis.
